# Volumetric MRI Analysis of a Case of Severe Ventriculomegaly

**DOI:** 10.3389/fnhum.2018.00495

**Published:** 2018-12-06

**Authors:** Gésine L. Alders, Luciano Minuzzi, Sachin Sarin, Benicio N. Frey, Geoffrey B. Hall, Zainab Samaan

**Affiliations:** ^1^Neuroscience Graduate Program, McMaster University, Hamilton, ON, Canada; ^2^Women’s Health Concerns Clinic, St. Joseph’s Healthcare, Hamilton, ON, Canada; ^3^Mood Disorders Program, Department of Psychiatry and Behavioural Neurosciences, McMaster University, Hamilton, ON, Canada; ^4^Developmental Neuroscience Laboratory, Department of Psychology, Neuroscience & Behaviour, McMaster University, Hamilton, ON, Canada

**Keywords:** ventriculomegaly, hydrocephalus, depression, ventricles, MRI, segmentation, neuroplasticity

## Abstract

We present a case of a 60-year-old male referred to a tertiary psychiatric facility for diagnostic assessment due to low mood and behavioral changes. Neurological examination of the patient was unremarkable. Magnetic resonance imaging (MRI) indicated overt ventriculomegaly with gross dilatation of lateral and third ventricles. Manual segmentation of gray matter, white matter and cerebrospinal fluid demonstrated that the patient had a ventricular volume almost 46 times greater than that of healthy volunteers in the same age range. Despite his striking degree of ventriculomegaly and cortical thinning, he presented primarily with psychiatric and cognitive complaints. These represented a major neurocognitive disorder. His behavior improved with a structured environment and routine instituted by the treating team. This is a dramatic example of the brain’s response to extreme structural remodeling. Elements of pluripotentiality may counteract degeneracy to preserve functions in cases of serious structural stress in the brain. Changes in the neural circuitry of emotional processing, and/or disruption in signaling pathways important for synaptogenesis may influence depression pathophysiology. How this circuitry is modified in cases of extreme structural stress such as long-standing overt ventriculomegaly, is unclear. This case demonstrates the ability of the brain to generate a normal phenotype despite structural changes that seem incompatible with advanced cognitive function, illustrating the substantial potential for adaptability and plasticity in the brain.

## Introduction

The idea of applying brain imaging to explore psychiatric disorders is not new (Andreasen, 1988); nonetheless, psychiatry has not yet found many clinical roles for neuroimaging. One of the challenges has been that although we can obtain detailed imaging of brain structure with a resolution of 0.8–1 mm, anatomical abnormalities are not specific, and it is often difficult to correlate them with brain function. Extreme cases of hydrocephalus that demonstrate this principle have been described in the literature (Lewin, 1980; Canu et al., 2005; Feuillet et al., 2007). Indeed, long-standing overt ventriculomegaly in adults has been proposed as a unique clinical entity, comprised of a form of chronic hydrocephalus that progresses without the clinical and behavioral symptoms that would be expected, given the often quite dramatic degree ventricular enlargement (Oi et al., 1996, 2000). The clinical manifestations of hydrocephalus depend on the time of appearance and nature of onset (Del Bigio, 2010). While some data gathered from rats and humans have suggested that the degree of ventricular dilatation may be associated with the degree of motor and cognitive deficits (Del Bigio et al., 2003; Olopade et al., 2012), this is not always the case (de Oliveira et al., 2011). Dr. John Lorber famously described a student with an IQ of 126 and an honors degree in mathematics, who was socially normal despite having massive hydrocephalus and only a thin mantle of cortical thickness (Lewin, 1980). Another described case is that of a 44-year-old married father of two who worked as a civil servant (Feuillet et al., 2007). Despite having severe hydrocephalus, he had an IQ of 75, verbal IQ of 84 and lived a relatively normal life; although, he did suffer from leg weakness, which had prompted his presentation. In the context of psychiatric disorders, it is important to identify any underlying organic causes.

Here, we present the case of a 60-year-old male who presented with mood symptoms and was referred to a tertiary psychiatric facility for diagnostic clarification and treatment recommendations for depressed mood. Interestingly, despite his striking degree of ventriculomegaly and cortical matter loss, he presented with few neurological findings and presented with primarily psychiatric and cognitive complaints.

This study was carried out in accordance with the recommendations of the Canadian Institutes of Health Research, Natural Sciences and Engineering Research Council of Canada, and Social Sciences and Humanities Research Council of Canada, Tri-Council Policy Statement: Ethical Conduct for Research Involving Humans. Written informed consent was provided by the substitute decision maker, providing consent for the publication of this report.

## Case Presentation

Patient CS, a single 60-year-old male presenting with a history of generalized anxiety with panic, major depressive disorder, and excessive guilt, was referred from a county hospital to a tertiary psychiatric facility for clarification of diagnosis and a more comprehensive assessment. His sister, and the family physician that had been following the patient for the past 4 years, helped provide collateral history. His family noted that he was born with a large head. He had a history of meningitis at the age of 9 or 10 after which it is thought that he developed a non-communicating hydrocephalus. His past psychiatric diagnoses included major depressive disorder, generalized anxiety disorder with panic, personality disorder, and “borderline intelligence.” He had several admissions to a psychiatric ward over the past 3 years for low mood and had been trialed on numerous psychotropic medications (citalopram, lithium carbonate, risperidone, olanzapine, quetiapine, paliperidone, clomipramine, clonazepam, lorazepam) with little effect or benefit. At the time of admission, he did not smoke, drink alcohol, or take illicit drugs. His past medical history was significant for hypothyroidism corrected with the use of thyroxine, bowel resections secondary to possible malignant changes, fatty liver with lobar resection secondary to liver cancer and nephrolithiasis.

He was born and raised in Europe until the age of 5, when he immigrated to Canada, and is bilingual. His family reported that he had always had a large head, micropenis, central obesity and short stature. He had a history of being bullied for “looking like a girl” and being different. At school his peers were physically aggressive, hitting him on his head. Born the youngest of seven siblings, he was raised by his parents and lived under their care into adulthood, until both parents passed away—his father had Diabetes Miletus and his mother had a brain tumor. Thereafter, he was taken care of by his sister. He had an older brother who also passed away secondary to a brain malignancy. One brother has dyslipidemia, and two sisters and one brother are healthy. He had no employment history and as a child had always struggled in school, completing a vocational stream of education until grade 10. Socially, he was active in a band for a few years (plays guitar well) and sang in a church choir. However, he never lived independently, and had no romantic relationships.

Initial assessment revealed that he was a poor historian unable to give an accurate timeline of events. He often expressed fears that he was going to die. He suffered from delusions of guilt that he had caused the deaths of family members. His conversation was repetitive, he repeatedly asked the same questions and restated his fear of dying despite several reassurances. He had no history of self-harm or suicide attempts. On physical examination, he had a wide stance waddling gait, slow movements, limited arm swing and masked facies. He was noted to have enlarged head circumference (62.5 cm) and limited insight into his illness and the need for treatment. His clinical presentation prompted examination with magnetic resonance imagining (MRI) of the brain and formal neuropsychological testing.

### Investigations

#### Magnetic Resonance Imaging

A sagittal T1, axial T2, axial T2 FLAIR and diffusion-weighted images were acquired throughout the brain. Findings indicated a long-standing overt ventriculomegaly, likely due to aqueductal stenosis, with bilateral gross dilation of the lateral and third ventricles, with a small aqueduct and fourth ventricle, with significant thinning of the corpus callosum and overlying cerebral cortex. Vascular flow-voids at the base of the brain were normal and there were no mass lesions, significant sulcal effacement, downward tonsillar herniation or restricted diffusion observed.

**Figure 1:**
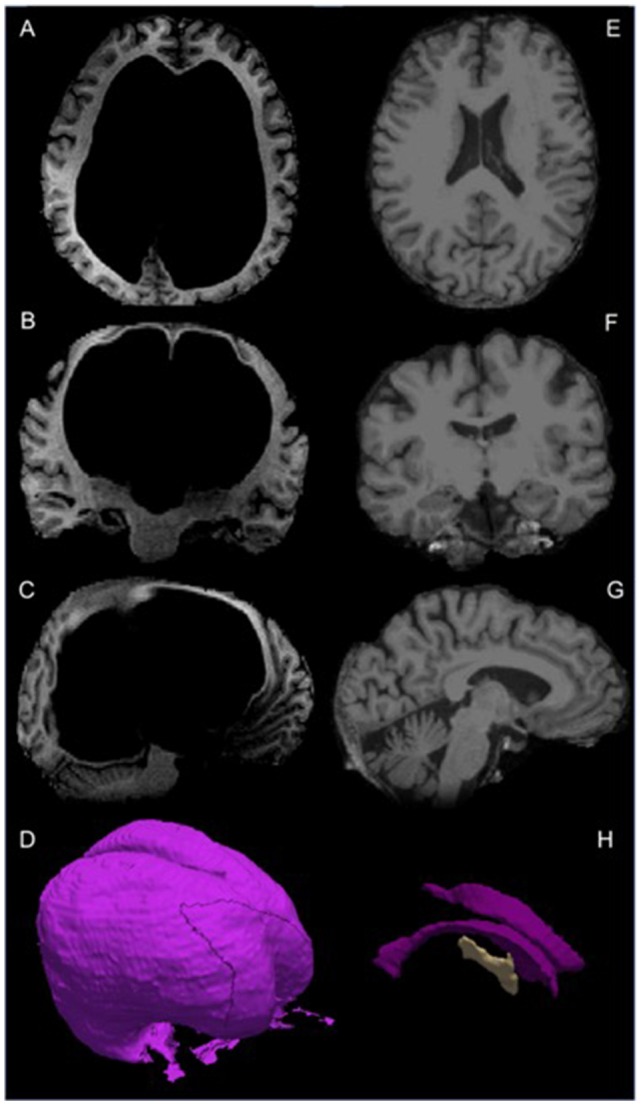
From top to bottom: T1-weighted magnetic resonance imaging (MRI) images in the transverse, coronal and sagittal planes of Patient CS **(A–C)** and a healthy age- and sex-matched control participant healthy control 1 (HC1) aged 60 **(E–G)**. Three dimensional image of Patient CS’ ventricular space **(D)**, and **(H)** three dimensional images of left and right lateral and third ventricles taken from the Freesurfer (Dale et al., 1999) average of 35 brains template.

Manual segmentation of gray and white matter and cerebrospinal fluid (CSF; Figure 1) of high-resolution T1 weighted MRI images was completed with Freeviewer in FSL (Jenkinson et al., 2012). Automatic segmentation of a comparison group of sex and age matched healthy controls (HCs; one aged 60, three aged 55 years, Table 1) was completed with the FreeSurfer (http://surfer.nmr.mgh.harvard.edu/) recon tool. The participant’s volumes were converted to *Z* scores for comparison. Compared to similarly aged control participants, the patient had extremely large ventricular volume (821,452 mm^3^, *Z* = 161), reduced white (333,606 mm^3^, *Z* = −2.655) and gray (432,184 mm^3^, *Z* = −3.07) matter volume, and within normal range total intracranial volume (1,587,242 mm^3^, *Z* = 0.57) see Table 1 and Figure 1.

**Table 1:** Comparison of ventricular volume, white matter, gray matter and total intracranial volume in patient CS compared to sex and age matched controls.

Participant (Age)	Ventricular volume* mm^3^ (%)	White matter volume** mm^3^ (%)	Gray matter volume*** mm^3^ (%)	Total estimated intracranial volume mm^3^
HC1 (60)	(18,642) 1.31%	(409,828) 29%	(563,787) 40%	1,419,546
HC2 (55)	(12,917) 0.91%	(418,055) 29%	(550,945) 39%	1,420,196
HC3 (55)	(24,599) 1.49%	(489,911) 30%	(670,738) 41%	1,648,324
HC4 (55)	(15,786) 0.99%	(492,760) 31%	(613,109) 39%	1,590,823
Means	(17,986) 1.18%	(452,639) 30%	(599,645) 39%	1,519,722
Patient CS	(821,452) 51.75%	(333,606) 21%	(432,184) 27%	1,587,242^#^
Patient CS’ Z scores	161.0	−2.655	−3.07	0.57

*Left and right lateral ventricles, left and right inferior lateral ventricles, third and fourth ventricles. **Left and right hemisphere cortical white matter. ***Subcortical gray matter, left and right hemisphere cortex, cerebellar gray matter. ^#^Total intracranial volume mm^3^.

#### Neurological Assessment

The patient’s neurological exam was unremarkable.

#### Neuropsychological Assessment

The Wechsler Adult Intelligence Scale (WAIS-III; Wechsler, 1997) revealed a borderline IQ of 79, with a verbal IQ of 88, non-verbal performance IQ of 74, poor working memory IQ of 71, verbal comprehension IQ of 93, and visual-spatial IQ of 80. The patient had difficulty completing tasks requiring working memory, which was in the 3rd percentile, and processing speed was extremely slow (in the 1st percentile). Hopkins Auditory Verbal Learning Test (Brandt, 1991) indicated severe memory impairment, with initial memory for only a few items, no significant recall between administrations, and inability to recall any information after a brief delay. Rey-Osterrieth Complex Figure Task (Osterrieth, 1944; Rey et al., 2014) performance indicated impaired visual spatial and working memory abilities with more attention to small details, missing elements and less attention to the overall image. The Stroop test (Stroop, 1935) indicated impaired executive function, scoring below the 1st percentile, with a severe inability to suppress automatic responses.

## Treatment

Patient CS was referred for a neurosurgery consult due to what appeared to be a long-standing history of hydrocephalus. The neurosurgery service recommended no role for neurosurgical intervention as there had been no recent decompensation of his chronic hydrocephalus. It was concluded that the patient’s increasing inability to cope at home and worsening cognitive ability represented an early onset major neurocognitive disorder. Interestingly, he may have suffered from panhypopituitarism that is known to be a rare exclusive presentation of chronic hydrocephalus (Edwards et al., 2004). Nonetheless, his sensory and motor deficits were noncontributory to his presentation compared to his mood and cognitive complaints.

While on the inpatient ward, his behavior improved in response to the structured environment and routine instituted by the treating team. In particular, he responded well to positive reinforcement and encouragement that reinforced positive behaviors, such as playing his guitar (including a 3-h jam session) and socializing with those around him. Furthermore, his rumination regarding guilt that he had somehow caused the death of his loved ones decreased when he was repeatedly given a rational explanation for their deaths. Treatment involved tapering the patient off most of his psychotropic medications. At discharge, the patient continued only on his thyroxine and cholestyramine as well as a small dose of citalopram due to patient’s and family’s preference, and their hopes that this would help reduce his anxiety symptoms.

## Discussion

This case provides a dramatic example of the brain’s adaptability in response to extreme structural remodeling and demonstrates one extreme of the clinical manifestations of long-standing hydrocephalus. What seems clear is that the brain has mechanisms in place for reorganization and preservation of function, such as redundancy or spared capacity (Lewin, 1980). This allows adaptability and functional reorganization of neural circuits, resulting in the retention of function. While the patient had ventricles almost 46 times greater than similar aged individuals, his gray and white matter volume, although appearing “compressed,” was, to a large extent, preserved. He was dependent, with some preserved functioning, no neurological complaints, presenting primarily with psychiatric and cognitive complaints. This may be influenced by structural relationships of pluripotentiality of the human brain (a one-to many structure-function relationship; Friston and Price, 2003), which may come at the cost of utilizing the cerebral reserve (Canu et al., 2005), and may be a reasonable explanation for his early onset of cognitive difficulties and perhaps a disposition (heightened vulnerability) for mood disturbances.

A recent review provides evidence for topographically organized interconnected networks between cerebellum, basal ganglia and the cortex, that span processing of cognitive, motor, and affective information (Bostan and Strick, 2018). This may be a network level example of degeneracy (Friston and Price, 2003)—“the ability of elements that are structurally different to perform the same function or yield the same output” (Edelman and Gally, 2001) and may explain some of the patient’s preserved function, including being fluent in two languages and mastering playing a musical instrument. Altered cortical mapping in congenitally blind humans suggests cortical regions are “cognitively pluripotent” during neurodevelopment, with regions associated with vision changing developmental trajectory to process information from different sensory modalities, shaped by experience and limited by physical connectivity (Bedny, 2017). Elements of pluripotentiality may counteract degeneracy to preserve functions in such cases of serious structural remodeling in the brain.

When we conceptualize psychiatric disorders, we believe that they originate in the brain and result from a complex interaction of genetic and environmental factors. However, understanding how psychiatric illness develops and manifests itself within the brain has proven to be far from a simple task. Convergent data from post-mortem studies and neuroimaging suggest that abnormalities in the neural circuitry underlying emotional processing play an important role in the pathophysiology of depression (Price and Drevets, 2010). Individuals who experience severe or prolonged stress are at an increased risk for neuropathological effects of stress including development of depression and other psychiatric disorders (Lucassen et al., 2014). Social and environmental risk factors, and adverse experiences, modulated by genetic factors, have been shown to impact the same neural circuits that underlie mood regulation (Meyer-Lindenberg and Tost, 2012). Further, disruption of principle signaling pathways important for synaptogenesis are associated with the pathophysiology of depression (Duman et al., 2016). While the precise mechanism of action of antidepressant treatments remains unclear, it has been suggested that they may work by enhancing neuronal plasticity (Castrén and Hen, 2013), providing one explanation for the delay between when treatment is initiated and when patients begin to experience symptom amelioration (Harmer and Cowen, 2013). It is through visualizing psychiatric disorders as disorders in neural circuitry that novel treatments like deep brain stimulation have been and continue to be developed (Lozano and Lipsman, 2013). Less invasive psychological and pharmacological interventions utilizing this model have yet to be developed. As this case demonstrates, the ability of the brain to generate a normal phenotype despite structural changes that seem incompatible with advanced cognitive function, illustrate the potential for adaptability and plasticity within the brain.

## Data Availability

The data for this manuscript are not publicly available because of privacy concerns. Requests to access anonymized data should be directed to ZS: samaanz@mcmaster.ca.

## Author Contributions

ZS, LM, BF and GH: study concept. GA and SS: manuscript first draft. GA, LM, SS, BF, GH and ZS: manuscript revisions. GA and LM: data analysis. GA: manual segmentation. All authors have read and approved the final manuscript.

## Conflict of Interest Statement

The authors declare that the research was conducted in the absence of any commercial or financial relationships that could be construed as a potential conflict of interest.
